# Comparative Assessment of Different Gold Nanoflowers as Labels for Lateral Flow Immunosensors

**DOI:** 10.3390/s21217098

**Published:** 2021-10-26

**Authors:** Nadezhda A. Taranova, Nadezhda A. Byzova, Svetlana M. Pridvorova, Anatoly V. Zherdev, Boris B. Dzantiev

**Affiliations:** Research Center of Biotechnology, A.N. Bach Institute of Biochemistry, Russian Academy of Sciences, Leninsky Prospect 33, 119071 Moscow, Russia; taranovana@gmail.com (N.A.T.); nbyzova@inbi.ras.ru (N.A.B.); sh-p_s@mail.ru (S.M.P.); zherdev@inbi.ras.ru (A.V.Z.)

**Keywords:** nanoparticles, nanoflowers, antibodies, immunochromatography, test strips, cardiac biomarkers

## Abstract

Many studies have found that gold nanoparticles with branched surfaces (nanoflowers) are markers for immunosensors that provide higher sensitivity gains than the commonly used spherical gold nanoparticles. Although the analytical characteristics of nanoparticle-using systems vary significantly depending on their size and shape, the question of choosing the best gold nanoflowers remains open. This work presents a comparative study of a panel of 33 preparations of gold nanoflowers formed by varying several parameters: the size of spherical nanoparticles-nuclei, the concentrations of nuclei, and tetrachloroauric acid during growth. The sizes of the resulting particles, their sorption capacity under antibody immobilization, mobility along membranes for lateral flow assays, and the effects of these parameters on the limits of detection of lateral flow immunoassay are characterized. The optimality of preparations obtained by growing a 0.2% *v*/*v* solution of nuclei with a diameter of 10 or 20 nm with tetrachloroauric acid at a concentration of 0.12 mM was shown. With their use, lateral flow immune tests were developed to determine markers of acute myocardial infarction—fatty acids binding protein and troponins I and T. The use of gold nanoflowers obtained under the proposed protocols led to significant gains in the limits of detection—3 to 10 times under visual detection and over 100 times under instrumental detection—compared to spherical gold nanoparticles. The significant increase under instrumental detection is due to the label’s low nonspecific binding.

## 1. Introduction

Various immunoanalytical and immunosensoric systems are widely used for medical and veterinary diagnostics, environmental monitoring, and consumer product control [1]. A significant widening of the variants of these systems and a lowering of their limits of detection (LODs) are notedly associated with the use of nanoparticles as reagent carriers and detectable markers [2]. Among various nanoparticles, gold nanoparticles are of particular interest because of the simplicity of their acquisition and varying properties, the possibilities for effective functionalization, and low detectable concentrations due to their unique plasmonic properties [3,4,5,6].

The benchmark for producing gold nanoparticles is the synthesis of gold nanospheres (GNSs) by citrate reduction of tetrachloroauric acid—the Turkevich–Frens method [7,8], which is still being actively studied [9,10]. However, for several analytical applications, it is preferable to use alternative gold nanoparticles—anisotropic or nonoriented, but with a developed surface for which various synthetic methods have been developed [11,12]. Recent studies have shown the advantages of the bioanalytical application of gold nanoflowers (GNFs), which are flower-like nanoparticles with a developed surface in the form of wavy or sharp protrusions (tips) [13,14,15,16].

The successful use of GNFs has been described in many lateral flow immunoassays (LFIAs), also known as immunochromatographic assays, an actively developing area of immunosensoric technologies. Immunochromatography is performed using test strips on which all analytical reagents are preapplied such that the liquid sample, after its contact with the test strip, moves along it under the action of capillary forces; this initiates analytical interactions and the formation of detectable colored zones [17]. This principle provides quick and easy-to-obtain assay results and determines the practical demand for LFIA tests [18]. Many studies have successfully used GNFs as antibody carriers and detectable markers for LFIA using decreased LODs, usually from 4 to 10 times compared to LFIAs using common GNSs [19,20,21,22,23]. However, most of these works were limited by the consideration of a single GNF preparation without substantiating the nanoparticle size and shape requirements and the grounded choice of a method for their synthesis. The exceptions are two works, where the series of GNFs obtained by varying either the nucleus size [16], or the tips length [24] were presented, and the LODs of test systems implemented using these GNFs were compared.

However, whether the optima in one parameter with other fixed synthesis conditions are absolute optima among the entire variety of GNFs is still unclear. Although some of the variable parameter combinations can be rejected at the GNF-obtaining stage because of the instability of their colloidal solutions, a significant number of variants remains possible. In addition, comparing GNFs solely by LOD for the LFIA of a particular antigen does not clarify the question of which analytically significant properties differ among various GNFs. The advantages of the proposed GNFs demonstrated for one case may not be retained when the GNFs are combined with other immune reactants or assay formats [25]. Therefore, a systematic comparison of GNFs obtained by varying different synthesis parameters is required, with an assessment of the intrinsic characteristics of GNFs and the achievable LODs of LFIAs. Therefore, this work contains a study of the effects of the GNF synthesis conditions (the size of GNS nuclei, the concentrations of the nuclei, and the tetrachloroauric acid used during the GNF-growing process), a comparison of physical properties, and the analytical characteristics of LFIAs for different GNFs and GNSs. Protocols for the optimal GNFs synthesis are proposed, which make it possible to increase the sensitivity of LFIA by a factor of 10–100, depending on the method of signal registration. The novelty of the work lies in the systematic study of the effect of the protocols for the synthesis of GNFs on their physical characteristics and, as a consequence, on the analytical parameters of immunoassay, which makes the choice of the optimal protocols more justified. The work included consideration of the use of conjugates of GNFs in the LFIA of three analytes, which indicates the universality of the established patterns.

The analytical systems characterized in this study cover LFIA tests for fatty acid-binding protein (FABP) and cardiac isoforms of troponins I (cTnI) and T (cTnT). Taking into consideration several immunoreagents’ combinations with different affinities and specificities makes it possible to assess the general nature of the patterns observed when comparing nanoparticles. The selected compounds belong to biomarkers released into the blood after an acute myocardial infarction and controlled during diagnosis [26,27]. Solutions for lowering their LODs, especially for troponins, help increase the reliability of emergency medical diagnostics and minimize false–negative test results [28,29].

## 2. Materials and Methods

### 2.1. Chemicals and Materials

Mouse monoclonal antibodies against cTnI, clones 19C7cc (Ab1/cTnI) and 4C2cc (Ab2/cTnI), cTnT, clones 7G7 (Ab3/cTnT) and 1F11 (Ab4/cTnT), FABP, clones 10E1 (Ab5/FABP) and 5B5 (Ab6/FABP), and recombinant antigens (i.e., FABP, troponin I, and troponin T) were from HyTest (Moscow, Russia). Goat antibodies against mouse immunoglobulins (GAMI) were from Arista Biologicals (Allentown, PA, USA). The absence of cross-reactions for the used monoclones with other proteins of blood was confirmed in our previous investigations [30,31]. The cardio marker-free serum TruLab N was from Diagnostic Systems (Holzheim, Germany).

Bovine serum albumin (BSA), tetrachloroauric acid (HAuCl_4_), tannic acid, sodium citrate, hydroquinone, Tween-20, Triton X-100, Tris, 3,3′,5,5′-tetramethylbenzidine dihydrochloride (TMB), poly(vinyl formal), sucrose, and sodium azide were from Sigma-Aldrich (St. Louis, MO, USA). Other chemicals (e.g., solvents, acids, salts) were from Chimmed (Moscow, Russia). All solutions for syntheses and assays were prepared using bidistilled water purified by the Sartorius Arium^®^ pro system (Göttingen, Germany). The water resistivity was no less than 18.2 MΩ cm.

To perform enzyme linked immunosorbent assays (ELISAs), Costar 9018 96-well polystyrene microplates (Corning Costar; Corning, NY, USA) were used. The lateral flow test strips were made from compounds of Mdi Easypack kits (Advanced Microdevices; Ambala Cantonment, India) membrane, which included a working nitrocellulose membrane CNPC with a 15 µm pore size, separation membrane GFB-R4, glass fiber membrane PT-R7, and absorption membrane AP045.

### 2.2. GNSs Synthesis

To obtain GNSs with a diameter of about 5 nm (as nuclei), HAuCl_4_ (5% *w*/*v*, 0.1 mL) was mixed with bidistilled water (39.5 mL), and heated at 60 °C. Then sodium citrate (1% *w*/*v*, 2.0 mL), tannic acid (1% *w*/*v*, 0.25 mL), K_2_CO_3_ (25 mM, 0.25 mL) and bidistilled water (7.5 mL) were added, and the prepared mixture was agitated, boiled for 30 min and then cooled [9]. The product was stored at 4 °C.

GNSs were prepared using the citrate method according to [10]. For GNSs with a diameter of about 10 and 20 nm (as nuclei) in 10 mL of a boiling aqueous solution of 0.01% *w*/*v* HAuCl_4_, 0.3 mL or 0.2 mL of a 1% *w*/*v* sodium citrate was added while stirring, respectively. For GNSs with a diameter of about 30 nm, a 1% *w*/*v* HAuCl_4_ (1.0 mL) was added to 97.5 mL of bidistilled water and boiled with followed addition of 1.5 mL of 1% *w*/*v* sodium citrate. The mixture was boiled for 25 min and then cooled. The product was stored at 4 °C.

### 2.3. GNF Synthesis

GNFs were synthesized using a modified technique of gold seed-mediated growth [11]. A solution of HAuCl_4_ in the final concentration from 0.01 to 0.6 mM, spherical gold seeds in the final content from 0.2 to 5.0% *v*/*v*, sodium citrate (1% *w*/*v*, 22 μL), and hydroquinone (300 mM, 100 μL) were added sequentially to bidistilled water (10 mL). The mixture was stirred at room temperature for 30 min. Finally, the obtained product was stored at 4 °C.

### 2.4. Characterization of Gold Nanoparticles

To implement transmission electron microscopy (TEM) gold nanoparticles solutions were applied to 300-mesh grids (Pelco International; Redding, CA, USA) coated with a poly(vinyl formal) film. Then the film was placed on the glass and exposed to 0.15% *v*/*v* solution of formvar in chloroform. The images were obtained with a JEM CX-100 microscope (Jeol; Tokyo, Japan) at 80 kV and analyzed by the Image Tool software (University of Texas Health Science Center, San Antonio, TX, USA).

Hydrodynamic size and ζ-potential of GNSs and GNFs were measured using a Zetasizer Nano (Malvern Pananlytical; Malvern, UK). The registration of dynamic light scattering was implemented at 25 °C for 10 s at a scattering angle of 173°.

The absorption spectra of the nanoparticles in were recorded using a Biochrom Libra S80 spectrophotometer (Biochrom; Cambridge, UK) in the wavelength range of 350–800 nm.

### 2.5. Synthesis of Antibody Conjugates with GNSs and GNFs

The optimal concentration of antibodies for immobilization on GNSs selected based on flocculation curves [32]. The antibodies were transferred to a 10 mM Tris buffer, pH 8.5 and the GNSs or GNFs (A_max_ = 1.0) solution was brought to pH 8.5 with 1 M potassium carbonate. A_max_ for GNSs was measured spectrophotometrically at 525 nm. A_max_ for GNFs was measured spectrophotometrically at the corresponding maximum absorption wavelength. Then, under vigorous stirring, antibodies were added in the following concentrations: 0.5–20 µg/mL for Ab6/FABP, 10 μg/mL for Ab4/cTnT and 10 μg/mL for Ab2/cTnI. The mixtures were incubated at room temperature for 15 min, after which 10% *w*/*v* BSA solution in water (*v*:*v* = 40:1) was added and incubated for 10 min under vigorous stirring. Conjugates were separated from unbound antibodies by centrifugation at 20,000× *g* for GNSs or 5000× *g* to 12,000× *g* for GNFs, depending on their size, at 4 °C for 15 min using a centrifuge Allegra 64R (Beckman Coulter; Indianapolis, IN, USA). The supernatant was then discarded and collected for further ELISA characterization. The precipitate was dissolved in 10 mM Tris-buffer, pH 8.5, with 1% *w*/*v* BSA and 1% *w*/*v* sucrose (TBSA) and centrifuged again under the same conditions. The resulting precipitate was dissolved in TBSA, after which sodium azide was added to the final concentration of 0.05% *w*/*v* and stored at 4 °C.

### 2.6. Determination of the Number of Antibodies in Conjugates by ELISA

The antigen was sorbed into the microplate wells from a solution with a 1 μg/mL concentration and incubated at 4 °C overnight. The microplate wells were washed 4 times to remove unbound molecules using PBS with 0.05% *v*/*v* Triton X-100 (PBST). The following solutions were then added to the microplate wells: (i) antibody solutions in the concentration range of 0–1000 ng/mL to obtain a calibration curve and (ii) supernatants after their separation by centrifugation in several dilutions to characterize the conjugates (see Section 2.5). The mixture was incubated for 1 h at 37 °C, and then the wells were washed 4 times with PBST. To the complexes formed in the wells, 50 μL of anti-species immunoperoxidase conjugate (dilution 1:3000 in PBST) was added, and this mixture was incubated for 1 h at 37 °C. After washing the microplate as described above, the peroxidase activity of the bound enzyme label was determined. To do so, 50 μL of the substrate mixture containing 0.4 mM TMB and 3 mM H_2_O_2_ in 40 mM sodium citrate buffer, pH 4.0, was added. After incubation at room temperature for 15 min, the reaction was stopped by adding 25 μL of 1 M H_2_SO_4_, and A_450_ was measured using a Zenyth 3100 microplate photometer (Anthos Labtec Instruments; Salzburg, Austria).

The number of bound antibodies was calculated using the linearization of the calibration curve based on the difference between the concentrations of the added antibodies and the antibodies found in the supernatant. Then, the obtained values were recalculated to determine the number of bound antibodies per nanoparticle.

### 2.7. Evaluation of Binding of Nanoparticle–Antibody Conjugates with Antigens (Functional Activity) by ELISA

The antigen was sorbed into the microplate wells, and unbound molecules were removed as described in Section 2.6. Then, 50 μL of GNFs–antibodies conjugates in PBST (A_max_ from 0.001 to 1.0) was added to the wells. The microplate was incubated for 1 h at 37 °C and then washed 4 times with PBST. Further stages were carried out as in Section 2.6. The obtained dependencies were used to determine the LODs.

### 2.8. Fabrication of Tests Strips for LFIAs

A working nitrocellulose membrane was processed using an IsoFlow dispenser (Imagene Technology; Lebanon, NH, USA). To form a control zone (CZ), a GAMI solution with a concentration of 0.5 mg/mL in PBS containing 0.25% *w*/*v* BSA, 0.25% *w*/*v* sucrose, and 0.1% *w*/*v* sodium azide was used. To form the test zone (TZ), solutions of Ab5/FABP (1.0 mg/mL), Ab1/cTnI (2.0 mg/mL), or Ab3/cTnT (2.0 mg/mL) in the same buffer were used. Of each solution, 32 μL was applied per 240 mm of the width of the sheet of the working membrane.

Antibody conjugates with gold nanoparticles were applied to the glass fiber membrane at dilutions corresponding to A_max_ = 4.0 for FABP, 5.0 for cTnI, or 2.0 for cTnT in TBSA buffer with 1% *v*/*v* Tween 20 in 400 μL per 240 mm membrane length. When preparing a series of test strips with a constant concentration of HAuCl_4_ equal to 0.12 mM (GNFs 20/0.12/0.2, 20/0.12/0.5, and 20/0.12/1, see below), the concentrations of the conjugates were calculated based on the minimum concentration of gold nuclei equal to 0.2% *v*/*v*. Other GNFs were normalized to A_max_.

The obtained sheets with applied reactants were assembled, including the separation and adsorption membranes, and were cut into strips 3.5 mm wide using an automatic guillotine Index Cutter-1 (A-Point Technologies; Gibbstown, NJ, USA).

After dispensing, all membranes were dried at room temperature for at least 20 h.

### 2.9. LFIA and Data Processing

LFIA was performed at room temperature. The test strip was immersed in a vertical position with its lower end for 1 min in an aliquot of the sample (model solution of the analyte or blood serum), after which it was placed on a horizontal surface. The intensity of TZ coloration was assessed after 10 min (or from 2 to 12 min with an interval of 30 s when studying the kinetics of the binding). The coloration was recorded using a CanoScan 9000F (Canon; Tokyo, Japan) scanner, after which the images were digitally processed by TotalLAB software (Cleaver Scientific; Rugby, UK).

The visual LOD (cutoff) was defined as a minimum analyte concentration at which the visually perceptible stained band was visible in the TZ. The instrumental LOD was defined as an analyte concentration at which the TZ staining intensity exceeded 3 times standard deviation for background staining of the TZ (samples without analyte).

## 3. Results

### 3.1. GNS Synthesis and Development of LFIA Test Systems Using GNSs

GNSs were obtained during the first stage of the analysis. On the one hand, they were used as nuclei in the two-stage GNF synthesis. Conversely, considering the characteristics of LFIA based on GNSs as the gold nanoparticles most widely used for analytical purposes will make it possible to adequately assess the capabilities and advantages of GNFs compared with GNSs.

Three GNS preparations were synthesized using the Turkevich–Frens method with varying citrate concentrations (see Materials and Methods). In addition, smaller GNSs were obtained by simultaneous reduction of HAuCl_4_ with tannic acid and sodium citrate. The sizes of all four preparations were characterized by TEM. According to the microphotography data, the average diameters of GNSs were 9.9 ± 3.8, 20.4 ± 1.1, and 29.3 ± 0.9 nm for the preparations obtained by the Turkevich–Frens method and 6.9 ± 1.4 nm for the preparation obtained using tannic acid. The shapes of the particles were close to spherical. Based on their rounded average diameters, these particles are indicated as GNSs 5 nm, GNSs 10, GNSs 20, and GNSs 30 nm, respectively. Table S1 contains a detailed characterization of the obtained preparations, including of GNFs.

GNSs 20 nm were conjugated with antibodies against the FABP, and GNSs 30 nm were conjugated with antibodies against the cTnI and cTnT. Ab6/FABP, Ab2/cTnI, and Ab4/cTnT were selected for conjugation from the available pairs of antibodies specific to each analyte. Based on previous studies’ data [30,31], these variants, in combination with the second antibodies immobilized in the TZ, provide more sensitive LFIAs. Choosing the antibody concentration used for immobilization on the GNSs 20 and 30 nm surfaces was based on the flocculation curves and the plateau points of these curves. It is known that the plateau of the flocculation curve reflects the stabilization of colloidal solutions by immobilized antibodies and a reduction in the free surface of gold in conjugates to a small extent, which excludes the nanoparticles aggregation at high ionic strength [32]. Generally accepted for GNSs used in LFIA, this criterion provides maximum sensitivity in sandwich assay schemes, although it may be accompanied by excessive consumption of antibodies [33]. A plateau was observed at an antibody concentration of 8 μg/mL for the flocculation curves obtained for all three preparations. Proceeding from these results and the literature recommendations [32], a 10–15% excess of antibodies was used for conjugation with respect to the plateau point: a concentration of 10 μg/mL (see Supplement Figure S1).

Based on the loss of antibody molecules from the solution during the immobilization course, it was shown that 65 ± 5% of the added antibodies are sorbed under the chosen conditions. When the conjugate solution was stored for 6 months at 4 °C, the degree of dissociation of the immobilized antibodies was insignificant—less than 5% of the bound amount.

Using conjugates of Ab6/FABP, Ab2/cTnI, and Ab4/cTnT with GNSs and Ab5/FABP, Ab1/cTnI, and Ab3/cTnT immobilized in the TZ, the three test systems based on 20 and 30 nm GNSs were optimized by varying the amounts of reagents applied to one test strip and finding the conditions that ensured reaching the minimum LOD. The chosen conditions are presented in Section 2 (Materials and Methods).

**Figure 1:**
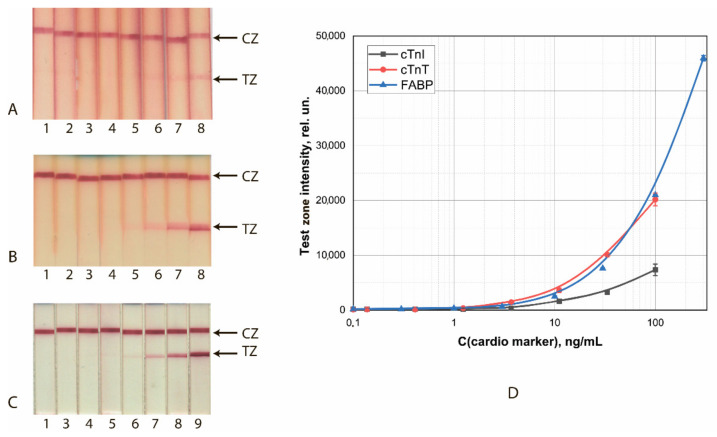
LFIA of cardio markers by test systems using the conjugates GNSs (30 nm)—Ab2/cTnI (**A**), GNSs (30 nm)—Ab4/cTnT (**B**), and GNSs (20 nm)—Ab6/FABP (**C**). (**A**–**C**) appearance of test strips after analysis of samples containing 0 (1), 0.1 (2), 0.4 (3), 1.2 (4), 3.4 (5), 11 (6), 33 (7), 100 (8), and 300 (9) ng/mL of the analytes. (**D**) Dependence of the intensity of coloration of the TZs on the concentration of antigen.

Calibration curves for analyte detection were obtained using the test strip series manufactured under the optimized conditions. The concentration of cardio markers varied from 0.3 to 300 ng/mL; the assay time was 10 min. The results of the detection of FABP, cTnI, and cTnT by test strips made using GNS conjugates are shown in Figure 1.

The achieved detection limits for visual (cutoff) and instrumental (LOD) detection are summarized in Table 1. With instrumental detection, all three systems clearly gave close (differing by no more than 2.5 times) LOD values.

### 3.2. Synthesis and Characterization of the GNFs’ Physical Parameters

**Figure 2:**
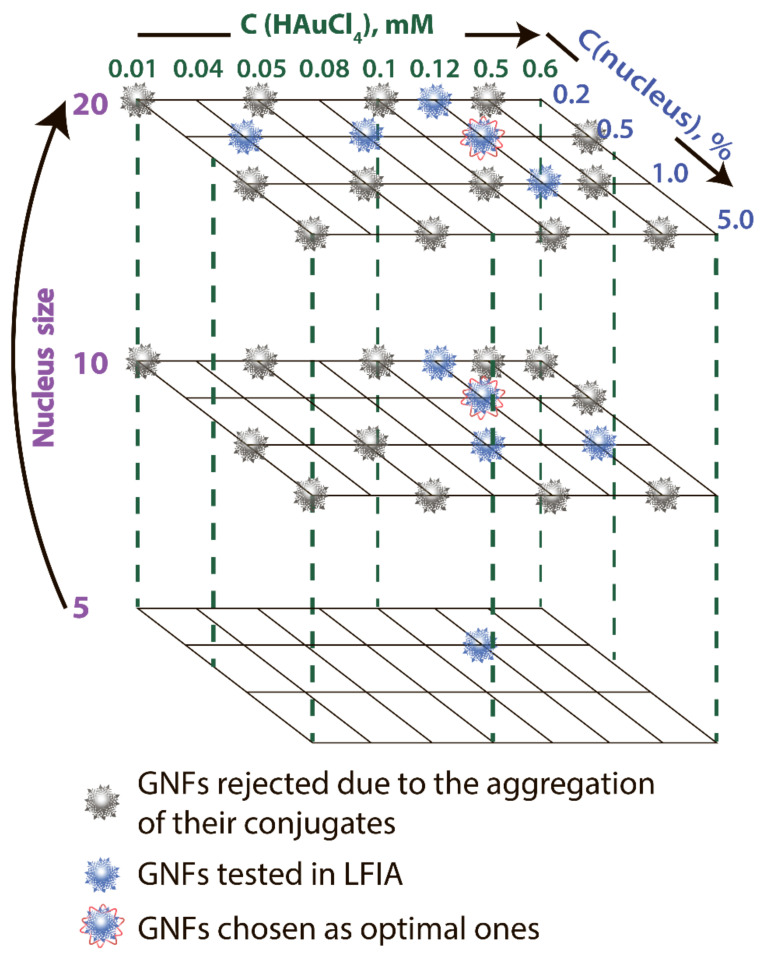
Variable parameters under GNF synthesis.

GNFs were synthesized by growing GNSs 5 nm, GNSs 10 nm, and GNSs 20 nm (nuclei) [11]. By varying the diameter of GNSs from 5 to 20 nm, the concentration of nuclei from 0.2 to 5.0% *v*/*v*, and the concentration of HAuCl_4_ from 0.01 to 0.6 mM, 33 GNF preparations were obtained. These preparations are indicated below in accordance with the three parameters: nucleus diameter, concentration of HAuCl_4_ in mM, and nucleus concentration in percentage. Decisions on the parameter variation range were made based on the protocols for the GNF synthesis presented in the literature. Figure 2 shows the variants used in the work, and Supplement Table S1 specifies detail composition, TEM sizes, DLS sizes, ζ-potential, and optical properties for some of these preparations. The reasons for choosing GNF for LFIA purposes are described below.

**Figure 3:**
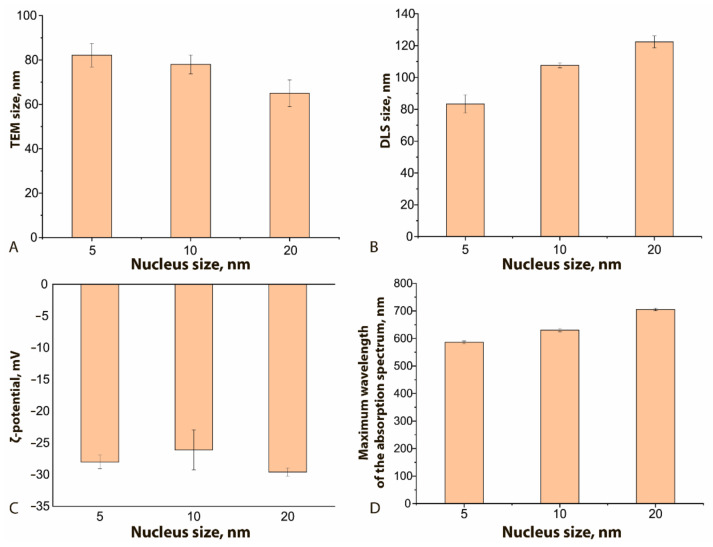
Influence of the nucleus size on GNFs’ physical characteristics: (**A**) TEM size, nm; (**B**) DLS size, nm; (**C**) ζ-potential, mV; (**D**) maximum wavelength, nm. C (HAuCl_4_) = 0.12 mM; C (nuclei) = 0.5% *v*/*v* for all samples.

Figure 3 demonstrates how the physical characteristics of the obtained GNFs differ for different nucleus sizes. As can be seen, the nucleus size has a different effect on the true diameter (according to TEM data, Figure 3A) from the resulting GNFs and their hydrodynamic diameter (Figure 3B). With an increase in this size from 5 to 20 nm, and with all other conditions of the synthesis being equal, the true diameter of nanoflowers decreased by 20%, and the hydrodynamic diameter increased by 32%. The first effect is likely due to a decrease in the number of nuclei per unit volume, whereas an increase in the hydrodynamic diameter can be interpreted as an increase in the size of hydration shells upon the formation of a more branched structure. The ζ-potential of the compared nanoflowers (Figure 3C) does not undergo significant changes and approaches −30 mV, which indicates the stability of the obtained colloidal solutions [34]. The maximum of the absorption spectrum with increasing nuclear size shifts to longer wavelengths, from 610 to 720 nm (Figure 3D), which correlates with similar effects for spherical nanoparticles [15].

**Figure 4:**
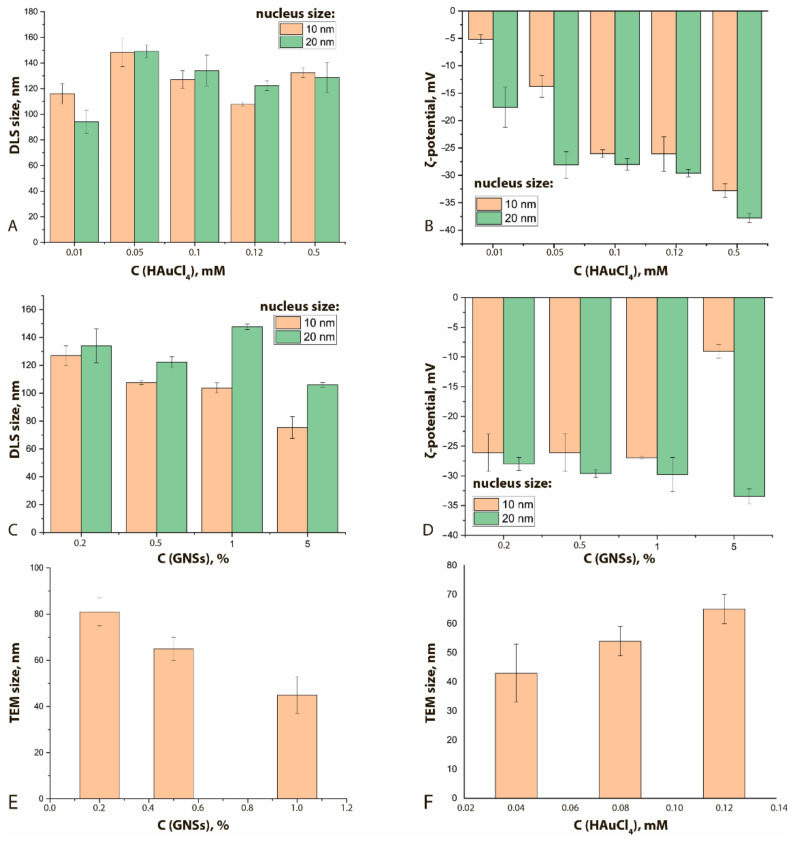
Influence of HAuCl_4_ concentration on the hydrodynamic diameter (**A**) and on ζ-potential (**B**) of GNFs (C(nucleus) = 0.2%). Influence of the concentration of nuclei (%) on the hydrodynamic diameters of GNFs (**C**) and on the ζ-potential (**D**) (C(HAuCl_4_) = 0.12 mM). Influence of the concentrations of nuclei (%, (**E**)) and HAuCl_4_ (mM, (**F**)) on the TEM-registered average diameters of GNFs (nucleus size 20 nm).

Comparisons of GNFs’ properties with other synthesis parameters are shown in Figure 4 and Supplementary Figure S4. An increase in the HAuCl_4_ concentration clearly leads to a nonmonotonic increase in the hydrodynamic diameters of GNFs (Figure 4A) and a decrease in their ζ-potential from −5 to −35 mV (Figure 4B). This effect can be explained by growth of tips of the GNFs with an increase of the HAuCl_4_ concentration at a constant nucleus concentration. The lack of new nucleation centers facilitates the reduction of Au^3+^ ions on the surface of already formed GNFs, thus forming sharp tips that have an uncompensated charge greater than that of rounded particles. To stabilize such particles in solution, water molecules must form large hydration shells, which leads to an increase in hydrodynamic size.

An increase in the nucleus concentration can lead to a decrease in the hydrodynamic diameter (Figure 4C) and an increase in the ζ-potential (Figure 4D). An increase in the concentration of nuclei (aggregation centers) at a constant concentration of HAuCl_4_ leads to the formation of a larger number of GNFs nanoparticles. In this case, smooth tips are formed, which are characterized by a more compensated surface charge.

In general, for the formed panel of preparations, with an increase in the concentration of nuclei from 0.2 to 1.0% *v*/*v*, the average true diameter of GNFs decreased almost twice, from 81 nm for GNFs 20/0.12/0.2 to 45 nm for GNFs 20/0.12/1 (Figure 4E,F), which can be explained by a reduction in the number of crystallization centers. The wide range of variation in repeated experiments is due to the irregular structure of GNFs. The changes in the hydrodynamic diameters of GNFs were oppositely directed for growing concentrations of nuclei and HAuCl_4_.

A pronounced form of GNFs was observed at nucleus concentrations in the range of 0.2 to 1.0% *v*/*v* and HAuCl_4_ in the range of 0.04 to 0.12 mM. A further increase in the HAuCl_4_ concentration led to the formation of ellipse-like structures.

Changing the shape and size of the particles led to a shift in the absorption peak (Table 2, Supplement Figures S2 and S3). With an increase in the HAuCl_4_ concentration, a shift of the absorption spectra maxima to longer wavelengths was observed, corresponding to a color change in the colloid from light blue to dark blue. This effect can be explained by the growth of the GNFs’ long pointed tips, which form a complex particle structure and reflect the long-wavelength region of the spectrum. With an increase in nucleus concentration from 0.2 to 1.0% *v*/*v*, the opposite effect was observed, accompanied by a color transition from blue to light purple. This occurrence can be explained by the fact that GNFs with small tips of a rounded shape were formed under the increase in the number of crystallization centers. Such particles are closer in shape to spherical ones and have wavelengths of the absorption peak in the range of 500–570 nm.

**Figure 5:**
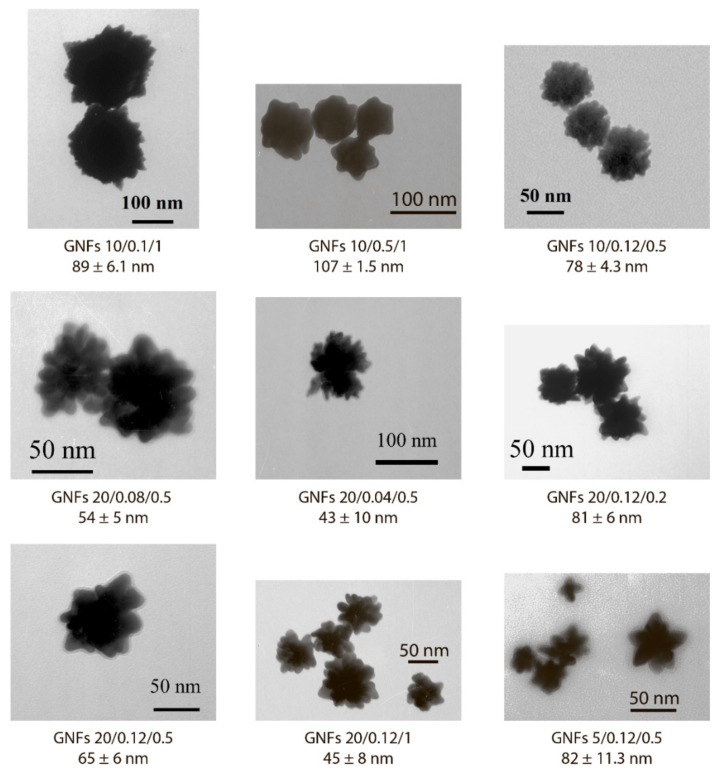
Appearance and dimensions of the chosen GNF preparations according to TEM data.

With an increase in the number of nuclei, the ζ-potential decreases; consequently, the stability of the preparations increases (Figure 5). Increasing the concentration of HAuCl_4_ also leads to an increase in the stability of the nanoflowers (Figure 4B). A change in the size of the nuclei does not cause significant shifts of their ζ-potentials.

Because the nonaggregation of GNFs is critical for further conjugation with antibodies and use in LFIA, 24 preparations characterized by low optical density (<0.2) and high ζ-potential (>−20 mV) were excluded from the panel. For the remaining 9 preparations, no precipitation was observed during the three-month storage, and the ζ-potential was in the range of −29.6 to −36 mV, which corresponds to the theoretical concepts and requirements for aggregation-stable solutions of nanoparticles [34]. The synthesis conditions for these nanoparticles are noted in Figure 2, and Figure 5 shows their average size and typical appearance.

### 3.3. Preparation of GNF Conjugates with Antibodies and Their Functional Characteristics

**Figure 6:**
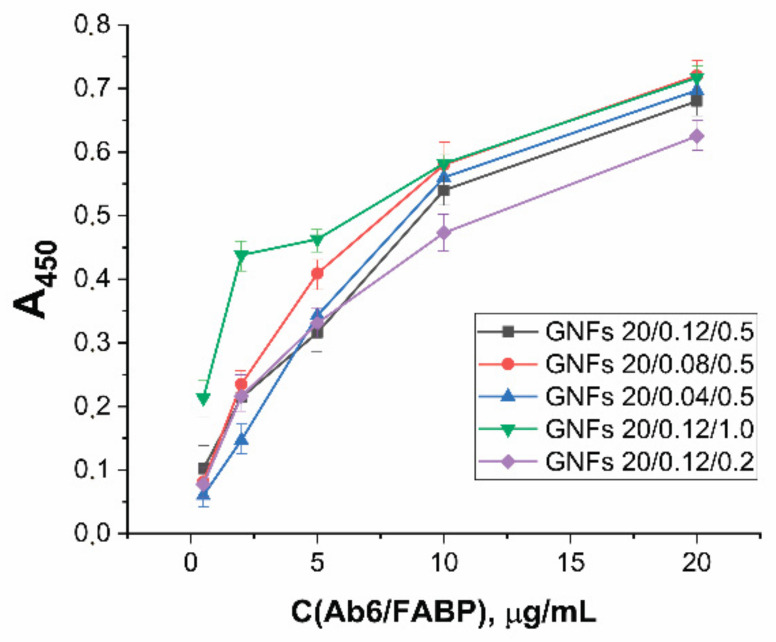
Dependence of optical density in ELISA tests of functional activities for GNFs–Ab6/FABP conjugates on Ab6/FABP concentration during conjugates syntheses for different GNFs.

For the selected GNF preparations, conjugates with antibodies specific to cardio markers were obtained. Unlike GNSs, GNFs have no literature recommendations for the choice of the number of immobilized antibodies that would provide the minimum LOD. The properties of conjugates with GNFs obtained at different ratio reagents were compared experimentally using antibodies to FABP as an example. The synthesis was carried out for GNFs (A = 1.0) and Ab6/FABP at concentrations from 0.5 to 20 μg/mL. Separated from unreacted antibodies, the resulting preparations were characterized by ELISA (see Section 2.7) for binding to FABP immobilized in microplate wells. The results obtained (Figure 6) indicate the concentration dependence did not reach saturation in any case. Over the entire range of antibody concentrations (up to a concentration of 20 μg/mL, twice as high as 10 μg/mL, selected for analogous synthesis with GNSs), an increase in antibody concentration was accompanied by an increase in conjugate binding.

**Figure 7:**
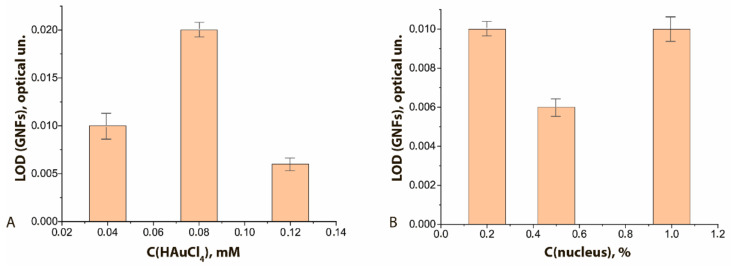
LODs of functional characterization of GNFs–Ab6/FABP conjugates by ELISA method: (**A**) influence of the C(HAuCl_4_); (**B**) influence of the C (nuclei). C(Ab6/FABP) was equal to 10 μg/mL.

The degree of binding differed for GNF preparations synthesized under different conditions. It was highest with maximal concentration of HAuCl_4_ in the reaction mixture (Figure 7A, see initial data in the Supplement Figure S5A–C) and had a pronounced optimum when the nucleus concentration was varied (Figure 7B, see initial data in the Supplement Figure S5A,D,E). In general, the higher sorption capacity of GNFs logically follows from their branched surface.

Additional experiments confirmed this interpretation to assess the ratio of antibody molecules binding during immobilization, which were carried out for Ab2/cTnI and Ab4/cTnT antibodies added to different GNFs at a fixed concentration of 10 ug/mL (as with GNSs). The methodology for measuring this parameter, which is based on a comparison of the amounts of added and unbound antibodies, is presented in Section 2.6; the results obtained are summarized in Table 3. Under the selected conditions, from 50% to 99% of the added Ab4/cTnT antibodies are sorbed, depending on the parameters characterizing the conditions for the GNF synthesis.

These levels are generally higher than antibody immobilization on GNSs under the same conditions (see Section 3.1). However, some antibody interactions with GNFs are reversible, as evidenced by a considerable decrease in the number of bound antibodies during storage of colloidal solutions of conjugates. For 2 months, for both the GNFs–Ab4/cTnT and GNFs–Ab2/cTnI conjugates, this decrease was 15%, which significantly exceeds the GNSs considered above. The observed difference logically followed from the GNFs’ developed and nonuniform curvature surface, which causes significant variation of sorption sites.

The results presented in this section indicate marked differences in the properties of antibody conjugates with different GNFs. However, the given differences do not allow for the unambiguous exclusion of some preparations from the consideration as less effective for LFIAs. The conditions for the interaction of immunoreagents during LFIA are significantly different for various reactants and their conjugates; for the formation of a detectable complex, the binding of a single antibody molecule on the surface of GNFs with an antigen in the sample is sufficient. Therefore, all the obtained GNF–antibody conjugates were further characterized from the viewpoint of their functioning in a lateral flow membrane system and of the achieved LODs.

### 3.4. Kinetics of the Movement of Nanoparticle Conjugates in a Lateral Flow on the LFIA Working Membrane

**Figure 8:**
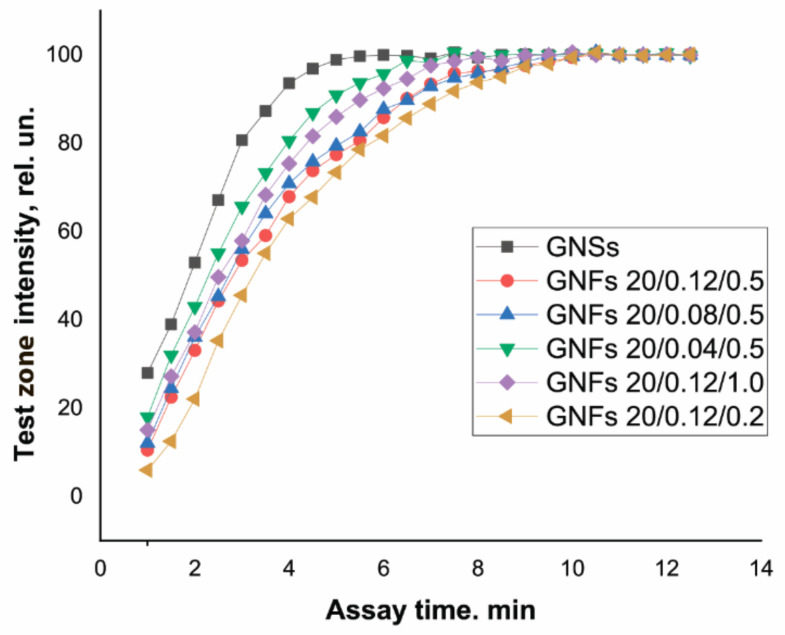
Dynamics of TZ coloration in LFIA test systems for FABP detection using conjugates of GNSs and GNFs (listed in the figure).

GNFs differ from GNSs in size and shape, which could affect the velocity of their movement along the test strip. This parameter is an important characteristic of immunoreagents that affects not only the time required to complete the analysis and obtain results but also the degree of approximation to the equilibrium of the reaction between the antigen in the sample and the antibody–nanoparticle conjugate during movement along the membrane and the quantity and composition of the resulting immune complexes. In this regard, the dynamics of color development in the TZ were studied using Ab6/FABP–antibody conjugates with GNSs and with different types of GNFs. The results obtained are presented in Figure 8.

The TZ coloration dynamics clearly depended on the size of the nanoparticles. Most rapidly, in 5 min, the coloration of the TZ reached its maximum when using GNSs with an average diameter of 20 nm. For GNFs, this time increased with increasing nanoparticle diameter, amounting to 7.5–8 min for particles with diameters of 43–45 nm (GNFs 20/0.04/0.5 and GNFs 20/0.12/1) and to 9.5–10 min for particles with diameters of 54 nm (GNFs 20/0.08/0.5) and 65 nm (GNFs 20/0.12/0.5).

Similar experiments were carried out for another pair of immunoreagents: cTnT and Ab4/cTnT. When using GNSs with a diameter of 30 nm, equilibrium was reached in 5 min; this process was the slowest for large (107 nm) GNFs 10/0.12/0.5, requiring 12 min to complete the color development. The slower movement of GNFs along the membrane compared to GNSs can be considered an advantage of using these nanoparticles as markers. An increase in the duration of the contact of the analyte with antibodies in the conjugate composition and in the immobilized TZ increased the number of formed and detectable labeled immune complexes, thereby reducing the LODs. Moreover, for all types of tested GNFs, the immunoreagent interaction was complete in a time not exceeding 15 min, that is, all variants of test systems can be qualified as rapid, as is true with GNSs.

### 3.5. Development and Characterization of GNF-Based LFIA Test Systems

The analysis conditions were optimized to ensure a correct comparative assessment of various GNF preparations in LFIA. This is similar to the aforementioned work for GNSs, with the same criteria for choosing the concentrations (numbers) of immunoreagents. The characteristics of the LFIAs of cardio markers considered below were obtained for the test systems manufactured in accordance with the found optimal parameters (see Section 2.8).

**Figure 9:**
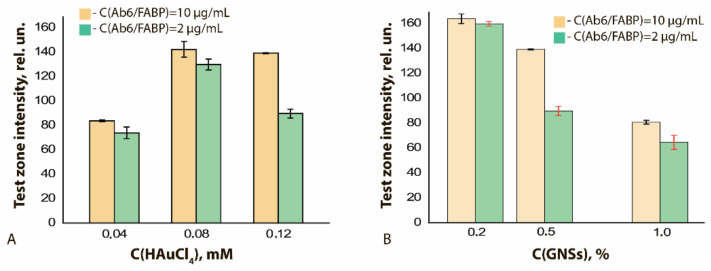
Dependences of the color intensity of the TZ for LFIA test strips for FABP after testing samples containing 300 ng/mL of FABP: (**A**) on the concentration of HAuCl_4_ in the reaction mixture at a constant concentration of nuclei (0.5% *v*/*v*); (**B**) on the concentration of nuclei at a constant concentration of HAuCl_4_ (0.12 mM).

Using GNFs differing in the nucleus and HAuCl_4_ concentrations used in their preparation, the concentration dependences of LFIA for FABP were obtained (see Figure S6). These dependences indicate the influence of both abovenamed concentrations on the LFIA parameters. At a constant concentration of nuclei in the reaction mixture equal to 0.5% *v*/*v* and an increase in the concentration of HAuCl_4_ from 0.04 to 0.12 mM (GNFs 20/0.04/0.5, GNFs 20/0.08/0.5, and GNFs 20/0.12/0.5, respectively), the degree of GNFs binding in the TZ increased 1.5 times. LOD varied from 4.0 to 1.5 ng/mL, and this effect persisted for different antibody concentrations (2 and 10 μg/mL; Figure 9A).

If the HAuCl_4_ concentration was fixed and equal to 0.12 mM, and if the nucleus concentration increased from 0.2% to 1.0% (GNFs 20/0.12/0.2, GNFs 20/0.12/0.5, and GNFs 20/0.12/1, respectively), then the marker binding in the TZ decreased by two times (from 160 rel. un. to 80 rel. un.), and the LOD of FABP changed from 11 to 1.5 ng/mL (Figure 9B). This effect can be explained by the fact that a decrease in the concentration of crystallization centers (nuclei) leads to the formation of larger particles detected in smaller amounts. The aforementioned TEM data for GNFs confirm this interpretation; the average diameters of GNFs in the series under consideration increased from 43 to 81 nm.

In addition to GNFs variants, conjugates that differed in the antibody concentration used for immobilization were compared. The corresponding concentration dependences of LFIA for FABP are shown in Figure S4. This study showed that an increase in the antibody concentration in the range of 0.5–20 μg/mL is accompanied by an increase in the coloration intensity of the TZ without reaching a plateau. However, the minimum cutoff of the assay results’ visual assessment is achieved at 10 μg/mL antibody concentration. The given cutoff is equal to 1.1 ng/mL of FABP.

The data set comparing LFIAs with various GNFs and their conjugates makes it possible to recommend GNFs synthesized using an HAuCl_4_ concentration of 0.12 mM and a nucleus concentration of 0.2% *v*/*v* to ensure the best LFIA sensitivity. The choice between nuclei of different diameters (10 and 20 nm) did not affect the characteristics of the test systems. These two optimal variants are marked in Figure 2.

**Figure 10:**
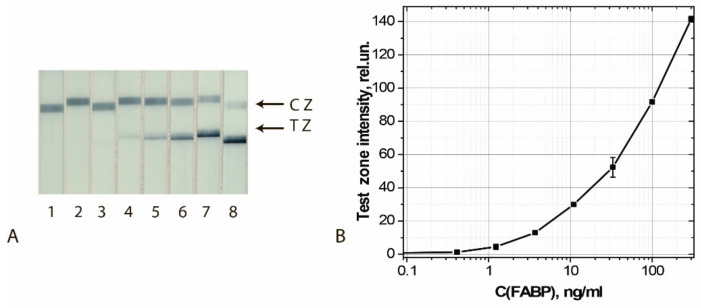
Detection of FABP in buffer by LFIA using the conjugate GNFs (20/0.12/0.2) Ab6/FABP. (**A**) Appearance of test strips after analysis of samples containing 0 (1), 0.4 (2), 1.2 (3), 3.7 (4), 11 (5), 33 (6), 100 (7), and 300 (8) ng/mL of FABP. (**B**) Dependence of the coloration intensity of the TZs on the concentration of FABP.

**Figure 11:**
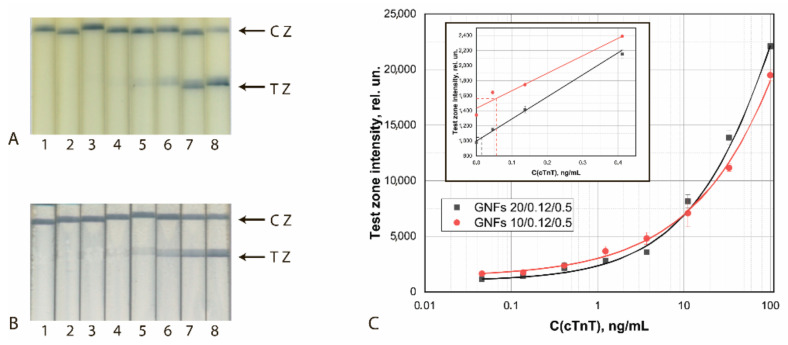
Detection of cTnT in buffer by LFIA using the conjugates GNFs (10/0.12/0.5)–Ab4/cTnT (**A**) and GNFs (20/0.12/0.2)–Ab2/cTnT (**B**). (**A**,**B**) Appearance of test strips after analysis of samples containing 0 (1), 0.12 (2), 0.4 (3), 1.2 (4), 3.7 (5), 11.1 (6), 33 (7), and 100 (8) ng/mL of cTnT. (**C**) Dependences of the coloration intensity of the TZs on the concentration of cTnT.

**Figure 12:**
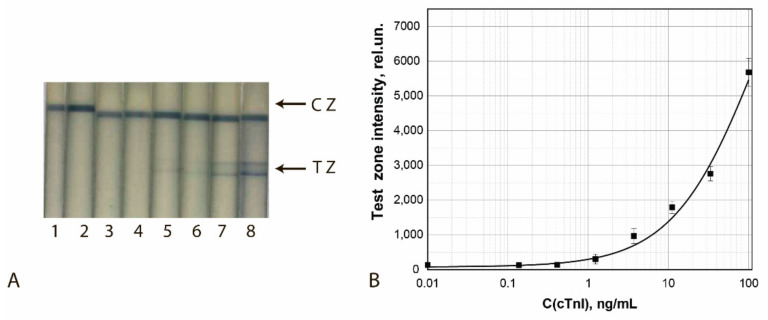
Detection of cTnI in buffer by LFIA using the conjugate GNFs (10/0.12/0.5)–Ab2/cTnI. (**A**) Appearance of test strips after analysis of samples containing 0 (1), 0.4 (2), 1.2 (3), 3.7 (4), 11.1 (5), 33.3 (6), 100 (7), and 1000 (8) ng/mL of cTnI. (**B**) Dependence of the coloration intensity of the TZs on the concentration of cTnI.

The results of the final approbation of LFIA test systems manufactured using the chosen best GNF preparations are shown for FABP, cTnT, and cTnI in Figure 10, Figure 11 and Figure 12, respectively. These results confirm the effectiveness of the proposed test strips’ completion when switching to real samples (blood serum).

Table 4 summarizes the analytical characteristics of GNS and GNF test systems. The transition from GNSs to GNFs makes it possible to reduce the limit of visual detection of analytes (cutoff) from 3 to 10 times and to reduce the limit of instrumental detection due to a lower background and higher signal amplitude up to 100 times.

Table 5 presents the published developments of LFIAs using GNFs and, if available, data on the comparisons performed between GNSs and GNFs. In our case, the gains achieved are close to the maximum, and the instrumental detection reflects the additional advantages of GNFs as markers reliably detected in LFIA in extremely low amounts. Furthermore, in the presented work, a systematic comparison of different GNFs and an assessment of their parameters affecting the assay were carried out for the first time. Combining these data will allow for reasonable selection of the best GNFs in the development of immunoassays for various new analytes.

The obtained and published data demonstrate the significant advantage of using GNFs as a marker for LFIA. This effect can be explained by the large size of GNFs and their larger area and highly developed surface, and, as a consequence, a large number of antibodies immobilized on their surface and a larger contact area in the flow. An important role in increasing the sensitivity is played by the slower motion of GNFs in the flow, which brings this system closer to equilibrium. An important fact is the high contrast of GNFs relative to GNSs.

## 4. Conclusions

The data presented in this work reflect the comparative capabilities of GNFs synthesized under different conditions as reagents for LFIA. They also show the synthesis-dependent and performance-influencing variation in the physicochemical properties of GNFs and the immunoreactivity of their antibody conjugates. Parallel consideration of the integration of GNFs with several combinations of immunoreagents of different specificities confirms the general nature of the established patterns. The optimal protocols for the synthesis of GNFs and their antibody conjugates proposed based on the study provide a 3- to 10-fold decrease in the LODs, as shown by the assays of three cardio markers, and a decrease up to 100 times for the limit of instrumental detection because of the low nonspecific binding.

## Figures and Tables

**Table 1:** Analytical characteristics of GNS-based test systems.

Analyte	Cutoff, ng/mL	LOD, ng/mL
FABP	11	1.4 ± 0.1
cTnT	11	2.0 ± 0.1
cTnI	33	3.5 ± 0.3

**Table 2:** Spectral characterization of the obtained GNFs.

Preparation	Absorption Peak, nm
GNFs 20/0.04/0.5	648–656
GNFs 20/0.08/0.5	690–702
GNFs 20/0.12/0.5	702–708
GNFs 20/0.12/0.2	771–776
GNFs 20/0.12/1.0	652–656

**Table 3:** Antibodies immobilized on the surface of gold nanoparticles by the adsorption method (for Ab4/cTnT as example).

GNFs	Immobilized Antibodies, %
10/0.1/1	89 ± 5
10/0.5/1	72 ± 7
10/0.5/0.2	90 ± 6
20/0.12/1	92 ± 4
20/0.04/0.5	78 ± 8
20/0.12/0.2	95 ± 6
10/0.12/0.5	99 ± 3
20/0.12/0.5	99 ± 4
5/0.12/0.5	50 ± 8
GNSs 30 nm	65 ± 5

**Table 4:** Analytical characteristics of test systems based on GNSs and GNFs.

Analyte	GNSs		GNFs (20/0.12/0.2)		GNFs (10/0.12/0.5)	
	Cutoff, ng/mL	LOD, ng/mL	Cutoff, ng/mL	LOD, ng/mL	Cutoff, ng/mL	LOD, ng/mL
FABP	11	1.4 ± 0.1	1.1	0.03 ± 0.008		
cTnT	11	2 ± 0.1	3.7	0.06 ± 0.01	1.2	0.01 ± 0.002
cTnI	33	3.5 ± 0.3			11	1.2 ± 0.1

**Table 5:** The use of GNFs in LFIA.

Particles	Size, nm	Assay Format	Analyte	GNFs vs. GNSs	Ref.
GNFs 20/0.3/1.2	80	Competitive LFIA	Clenbuterol	5 times >	[19]
GNFs 5/0.2/0.4	37.7	Competitive LFIA	Four mycotoxins	-	[35]
GNFs 20/0.18/0.5	100	Sandwich LFIA	Procalitonion	5 times >	[20]
GNFs 20/0.18/2.1	55	Sandwich LFIA	Cancer marker	-	[36]
GNFs 18/0.3/1.78	80	Competitive LFIA	Zearalenone	-	[37]
GNFs 40/0.25/2	80	Competitive LFIA	Ochratoxins A and B	5 times >	[21]
GNFs 20/0.18/0.75	80	Competitive LFIA	Cd^2+^	12 times >	[22]
GNFs 40/0.25/2	79	Competitive LFIA	Lactoferrin	4 times >	[23]
GNFs 20/0.09/2.5	35	Competitive LFIA	Ochratoxin A	4 times >	[24]
GNFs 3.5/0.2/0.4	33	Sandwich LFIA	Chorionic gonadotropin	-	[16]
20/0.3/4	47				
66/0.3/2.5	194				
GNFs 20/0.12/0.5	65	Sandwich LFIA	Cardio markers	3–10 times >	This work
10/0.12/0.5	78				

## Data Availability

The data presented in this study are available on request from the corresponding author.

## Supplementary Materials

The following are available online at https://www.mdpi.com/article/10.3390/s21217098/s1, Figure S1: Absorption curves for antibodies, Figure S2: Panel of synthesized GNFs preparations, Table S1: Physical characteristics of gold nanoparticle preparations, Figure S3: Spectra for GNFs (l = 2 mm), Figure S4: DLS data for GNFs, Figure S5: Concentration dependences of conjugate binding: GNFs 20/0.12/0.5–Ab6/FABP (A), GNFs 20/0.08/0.5–Ab6/FABP (B), GNFs 20/0.04/0.5–Ab6/FABP (C), GNFs 20/0.12/1.0–Ab6/FABP (D) и GNFs 20/0.12/0.2–Ab6/FABP (E) with FABP immobilized on the surface of microplate. Curves correspond to concentrations Ab6/FABP, equal 20 (1), 10 (2), 5 (3), 2 (4) и 0.5 (5) µg/mL, Figure S6: Concentration dependences of FABP detection by LFIA test systems with conjugates GNFs 20/0.12/0.5–Ab6/FABP(0.5–20) (A), GNFs 20/0.08/0.5–Ab6/FABP(0.5–20) (B), GNFs 20/0.04/0.5–Ab6/FABP(0.5–20) (C), GNFs 20/0.12/1–Ab6/FABP(0.5–20) (D) и GNFs 20/0.12/0.2–Abs/FABP(0.5–20) (E). Curves correspond to concentrations Ab6/FABP in the synthesis of conjugates equal to 0.5 (1), 2 (2), 5 (3), 10 (4) и 20 (5) µg/mL.
