# Supplementary material for: Comparative Assessment of Different Gold Nanoflowers as Labels for Lateral Flow Immunosensors
Source: Sensors (Basel). 2021 Oct 26;21(21):7098. doi: 10.3390/s21217098 (PMC8587648; doi:10.3390/s21217098)
Supplement: Supplementary file 1 [file sensors-21-07098-s001.zip › sensors-1432717-supplementary.pdf]

Supplementary Materials

Comparative assessment of different gold nanoflowers as labels for lateral flow immunosensors

Nadezhda A. Taranova, Nadezhda A. Byzova, Svetlana M. Pridvorova, Anatoly V. Zherdev and Boris B. Dzantiev*

A.N. Bach Institute of Biochemistry, Research Center of Biotechnology of the Russian Academy of Sciences, Leninsky prospect 33, 119071 Moscow, Russia

*Corresponding author: Boris B. Dzantiev

E-mail: dzantiev@inbi.ras.ru

Tel. +7 (495) 954-31-42

Fax +7 (495) 954-28-04

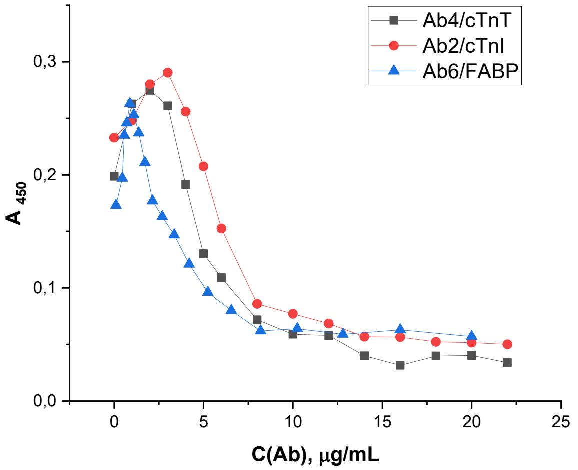

Figure S1. Absorption curves for antibodies.

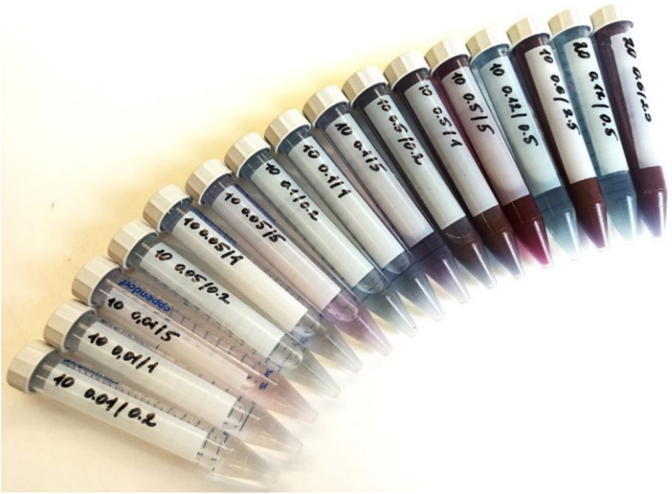

Figure S2. Panel of synthesized GNFs preparations.

Table S1. Physical characteristics of gold nanoparticle preparations

N	Preparation	Nuclei diameter, nm	$HAuCl_4$ concentration in final mixture, nM	Nuclei concentration in final mixture, nM	Spectral peak, nm	Diameter (TEM data), nm	Diameter (DLS data), nm	ζ-potential, mV
1	GNSs 5 nm				516	7 ± 1.4	12 ± 0.2	
2	GNSs 10 nm				518	10 ± 3.8	24 ± 0.6	-43.3 ± 1.8
3	GNSs 20 nm				520	20 ± 1.1	31 ± 2.7	-35.8 ± 2.4
4	GNSs 30 nm				525	29 ± 0.9	35 ± 1.0	-38.6 ± 1.0
5	GNFs 10/0.01/0.2	10	0.01	0.2	540		116 ± 7.9	-5.2 ± 0.8
6	GNFs 10/0.01/1	10	0.01	1	530		112 ± 10.9	-6.7 ± 4.5
7	GNFs 10/0.01/5	10	0.01	5	540		70 ± 14.3	-10.5 ± 11.6
8	GNFs 10/0.05/0.2	10	0.05	0.2	540		148 ± 11.0	-13.8 ± 2.0
9	GNFs 10/0.05/1	10	0.05	1	543		117 ± 4.0	-15.6 ± 1.0
10	GNFs 10/0.05/5	10	0.05	5	545		90 ± 4.4	-28.7 ± 0.4
11	GNFs 10/0.1/0.2	10	0.1	0.2	630		127 ± 7.0	-26.0 ± 0.7
12	GNFs 10/0.1/1	10	0.1	1	600	89 ± 6.1	104 ± 3.7	-27.0 ± 0.2
13	GNFs 10/0.1/5	10	0.1	5	550		75 ± 7.8	-9.1 ± 1.2
14	GNFs 10/0.12/0.5	10	0.12	0.5	630	78 ± 4.3	108 ± 1.6	-26.1 ± 3.2
15	GNFs 10/0.5/0.2	10	0.5	0.2	625		133 ± 3.8	-32.8 ± 1.3
16	GNFs 10/0.5/1	10	0.5	1	605	107 ± 1.5	115 ± 4.2	-33.0 ± 0.2
17	GNFs 10/0.5/5	10	0.5	5	550		79 ± 3.0	-32.7 ± 0.9
18	GNFs 10/0.6/2.5	10	0.6	2.5	575		102 ± 4.1	-33.0 ± 1.0
19	GNFs 20/0.01/0.2	20	0.01	0.2	710		94 ± 9.2	-17.6 ± 3.6
20	GNFs 20/0.01/1	20	0.01	1	710		102 ± 6.7	-14.8 ± 3.4
21	GNFs 20/0.01/5	20	0.01	5	504		51 ± 4.4	-28.9 ± 3.0
22	GNFs 20/0.05/0.2	20	0.05	0.2	710		149 ± 4.8	-28.1 ± 2.4
23	GNFs 20/0.05/1	20	0.05	1	710		110 ± 2.8	-31.1 ± 1.0
24	GNFs 20/0.05/5	20	0.05	5	710		154 ± 11.0	-0.4 ± 0.4
25	GNFs 20/0.08/0.5	20	0.08	0.5	696	54 ± 5.2		
26	GNFs 20/0.04/0.5	20	0.04	0.5	652	43 ± 10.3		
27	GNFs 20/0.1/0.2	20	0.1	0.2	710		134 ± 12.1	-28.0 ± 1.1

28	GNFs 20/0.1/1	20	0.1	1	630	39 ± 1.0	148 ± 2.0	-29.8 ± 2.9
29	GNFs 20/0.1/5	20	0.1	5	580		106 ± 1.7	-33.5 ± 1.3
30	GNFs 20/0.5/0.2	20	0.5	0.2	590		129 ± 11.7	-37.8 ± 0.9
31	GNFs 20/0.5/1	20	0.5	1	620	31 ± 2.7	134 ± 6.9	-36.6 ± 1.9
32	GNFs 20/0.5/5	20	0.5	5	545		123 ± 3.0	-34.1 ± 1.4
33	GNFs 20/0.12/0.2	20	0.12	0.2	773	81 ± 6.4		
34	GNFs 20/0.12/0.5	20	0.12	0.5	705	65 ± 6.0	122 ± 3.8	-29.6 ± 0.7
35	GNFs 20/0.12/1	20	0.12	1.0	654	45 ± 8.2		
36	GNFs 20/0.6/2.5	20	0.6	2.5	590		112 ± 4.3	-28.9 ± 0.4
37	GNFs 5/0.12/0.5	5	0.12	0.5	586	82 ± 11.3	83 ± 5.8	-28.0 ± 1.1

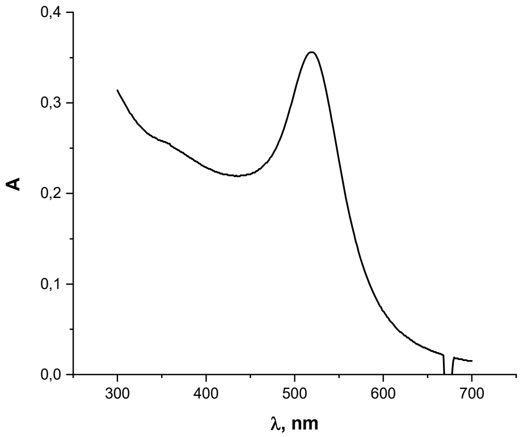
0,4
0,3
0,2
0,1
0,0
A
300
400
500
600
700
λ, nm

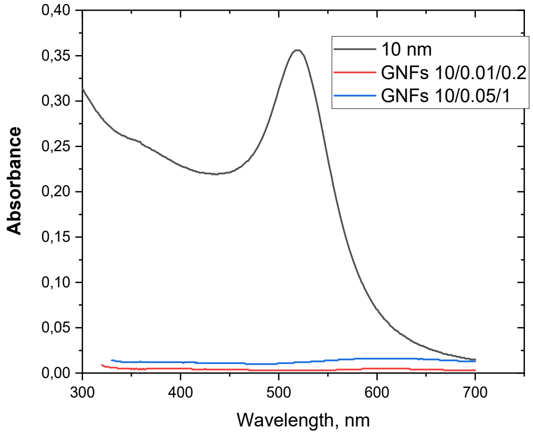
10 nm
GNFs 10/0.01/0.2
GNFs 10/0.05/1
Absorbance
Wavelength, nm

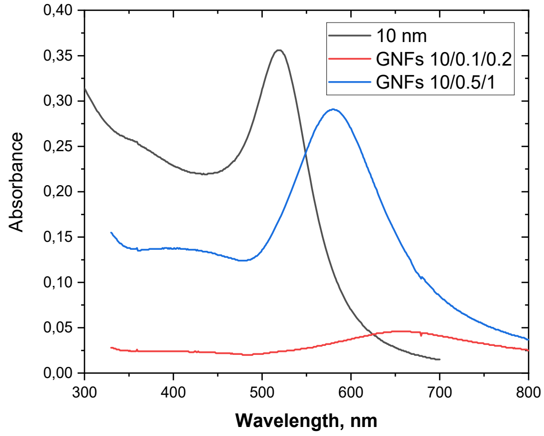
10 nm
GNFs 10/0.1/0.2
GNFs 10/0.5/1
Absorbance
Wavelength, nm

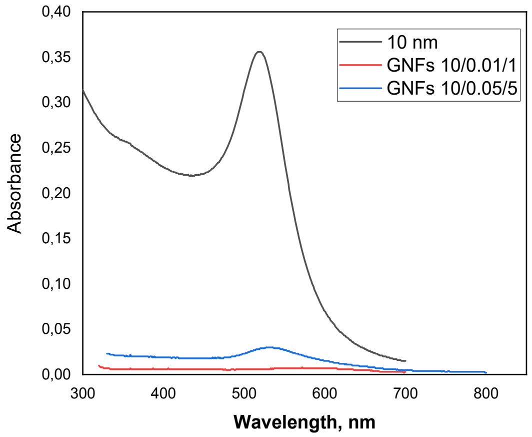
10 nm
GNFs 10/0.01/1
GNFs 10/0.05/5
Absorbance
Wavelength, nm

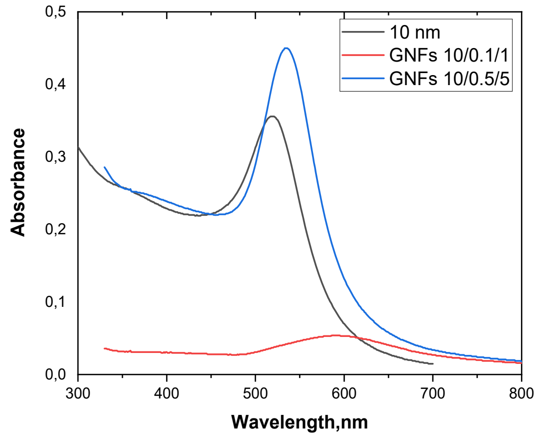
10 nm
GNFs 10/0.1/1
GNFs 10/0.5/5
Absorbance
Wavelength,nm

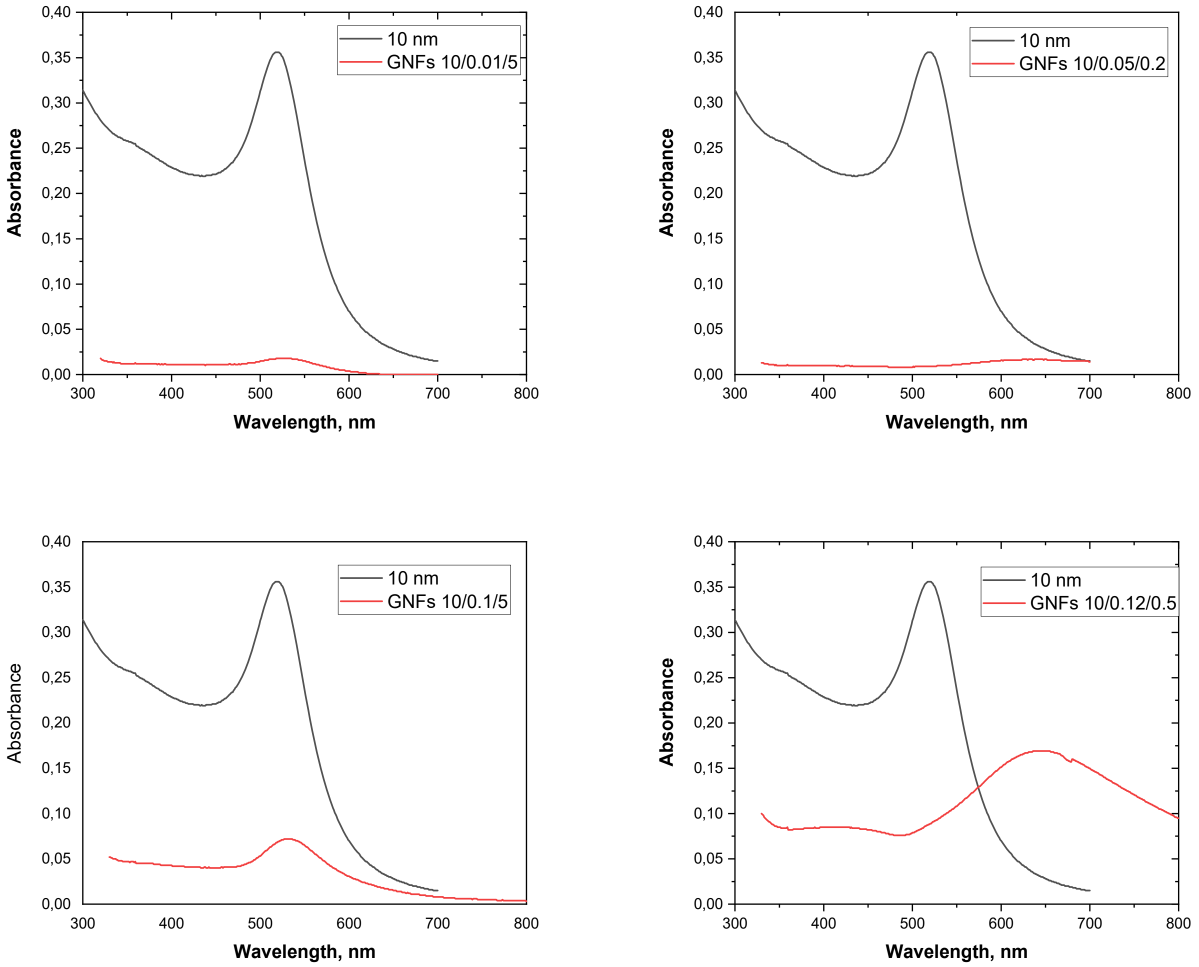

Figure S3. Spectra for GNFs (optical path length was 2 mm)

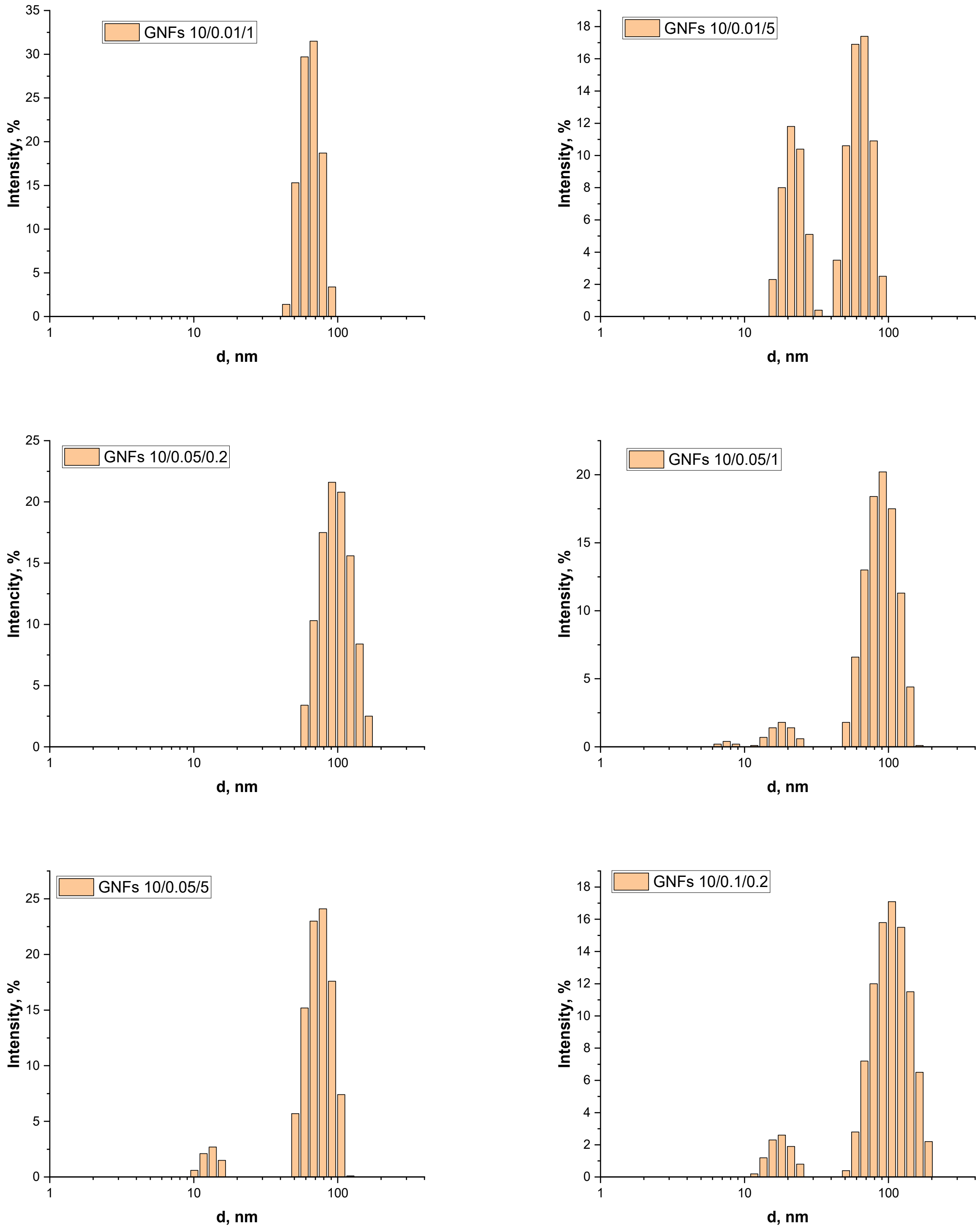

GNFs 10/0.01/1
GNFs 10/0.01/5
GNFs 10/0.05/0.2
GNFs 10/0.05/1
GNFs 10/0.05/5
GNFs 10/0.1/0.2
Intensity, %
Intencity, %
d, nm

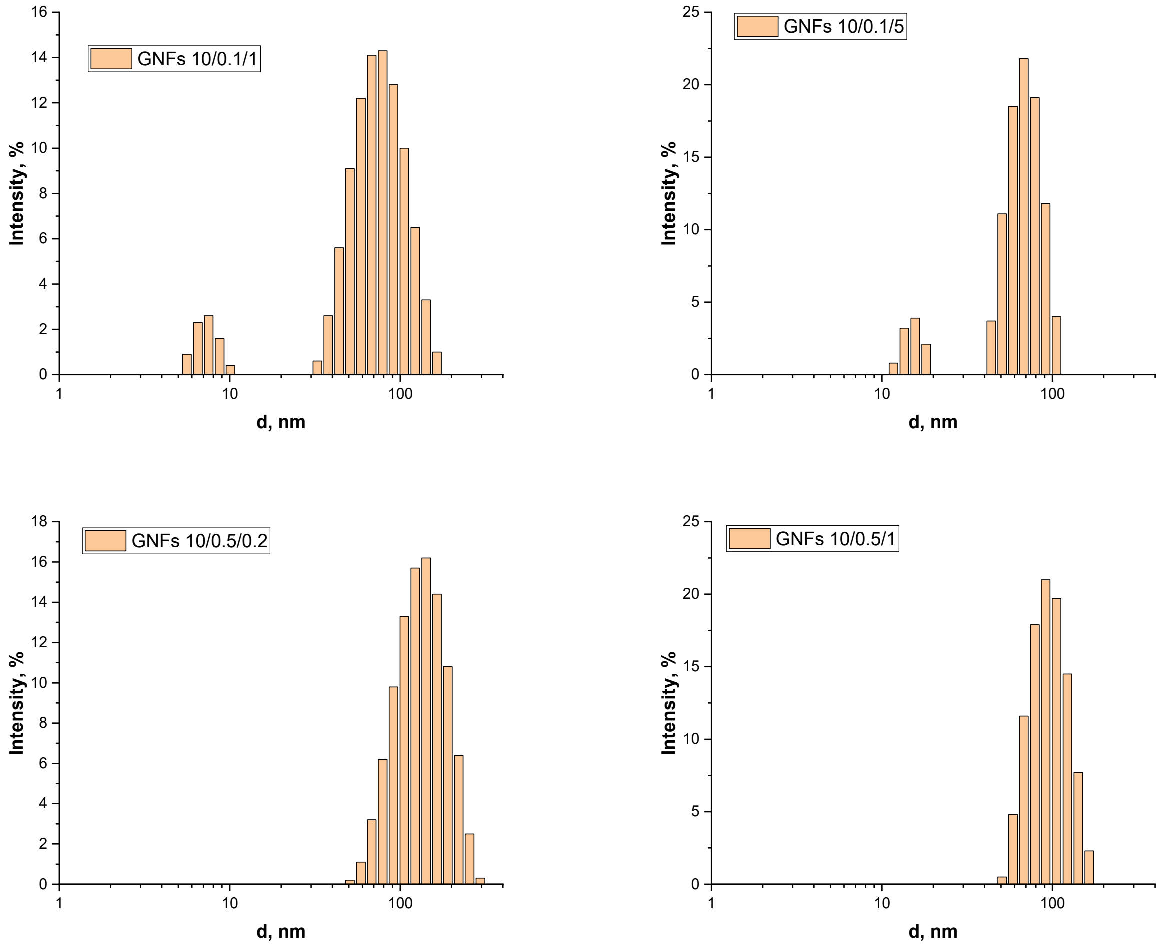
GNFs 10/0.1/1
GNFs 10/0.1/5
GNFs 10/0.5/0.2
GNFs 10/0.5/1
Intensity, %
d, nm

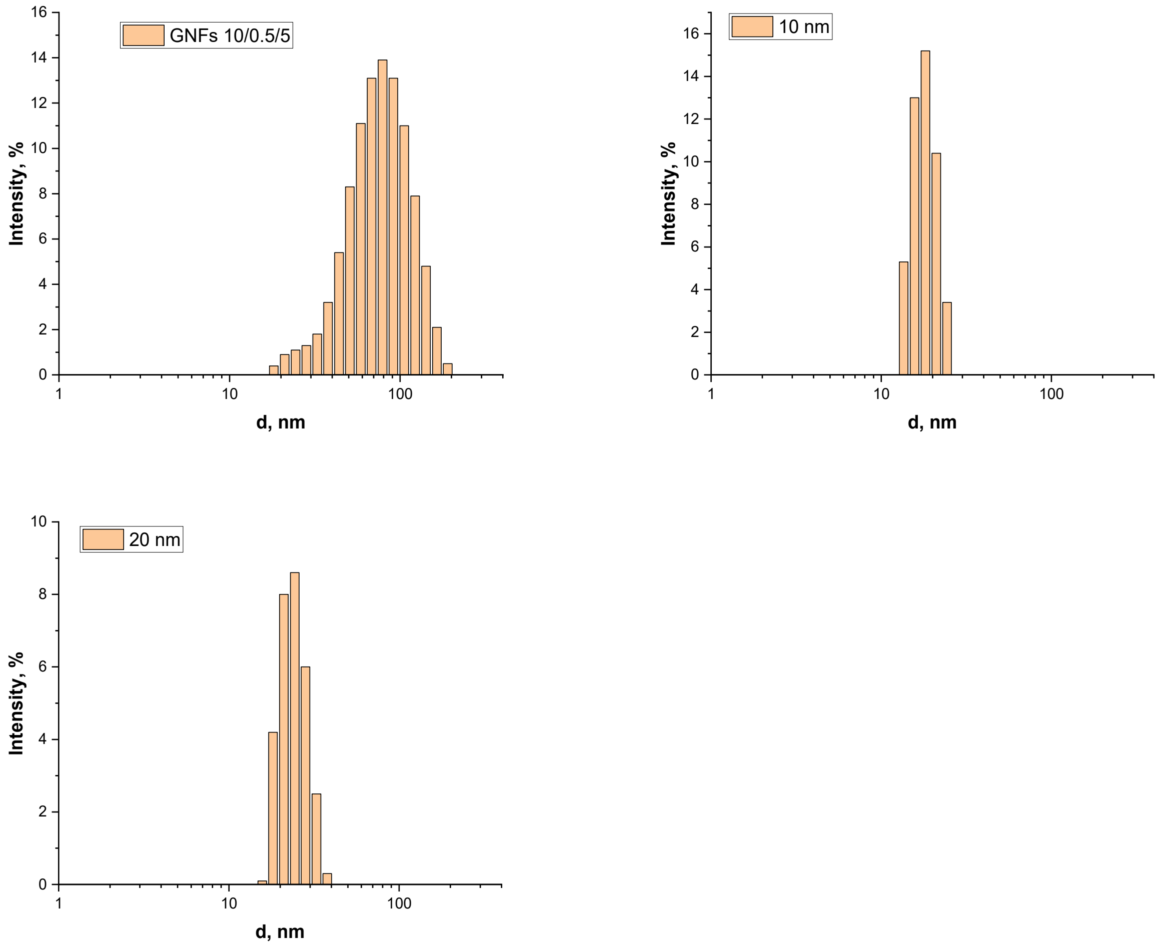

Figure S4. DLS data for GNFs

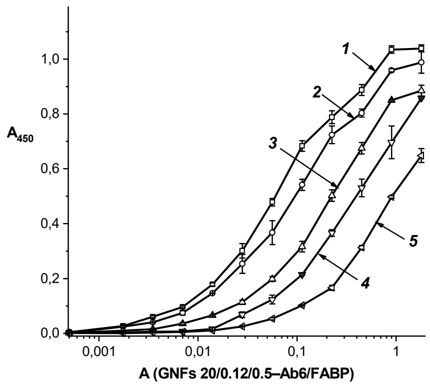
A450
1,0
0,8
0,6
0,4
0,2
0,0
0,001
0,01
0,1
1
1
2
3
4
5
A (GNFs 20/0.12/0.5–Ab6/FABP)

A

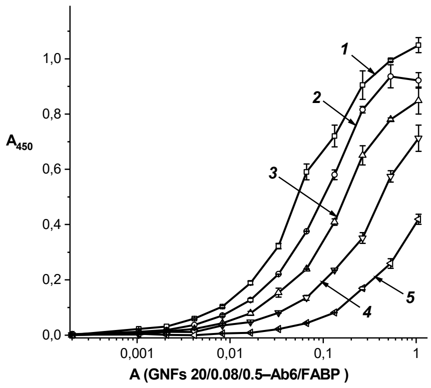
A450
1,0
0,8
0,6
0,4
0,2
0,0
0,001
0,01
0,1
1
1
2
3
4
5
A (GNFs 20/0.08/0.5–Ab6/FABP)

B

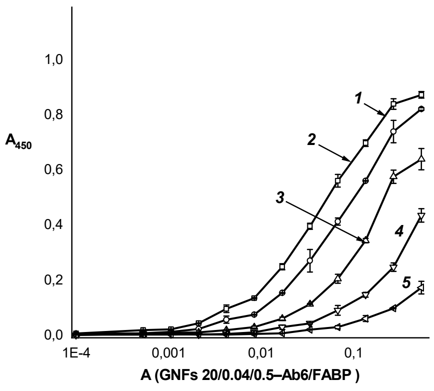
A450
1,0
0,8
0,6
0,4
0,2
0,0
1E-4
0,001
0,01
0,1
1
2
3
4
5
A (GNFs 20/0.04/0.5–Ab6/FABP)

C

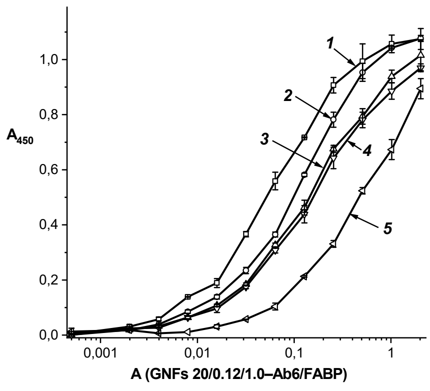
A450
1,0
0,8
0,6
0,4
0,2
0,0
0,001
0,01
0,1
1
1
2
3
4
5
A (GNFs 20/0.12/1.0–Ab6/FABP)

D

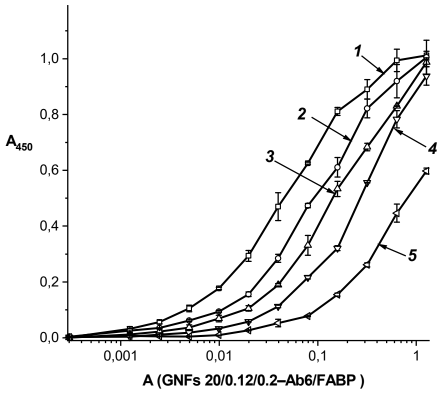

E

Figure S5 – Concentration dependences of conjugate binding: GNFs 20/0.12/0.5–Ab6/FABP (A), GNFs 20/0.08/0.5–Ab6/FABP (B), GNFs 20/0.04/0.5–Ab6/FABP (C), GNFs 20/0.12/1.0–Ab6/FABP (D) и GNFs 20/0.12/0.2–Ab6/FABP (E) with FABP immobilized on the surface of microplate. Curves correspond to concentrations Ab6/FABP, equal 20 (1), 10 (2), 5 (3), 2 (4) и 0.5 (5) μg/mL

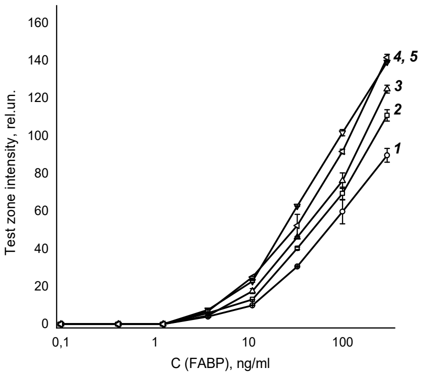

Test zone intensity, rel.un.
C (FABP), ng/ml
4, 5
3
2
1

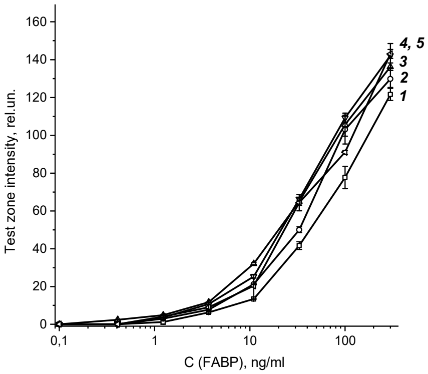

Test zone intensity, rel.un.
C (FABP), ng/ml
4, 5
3
2
1

A

B

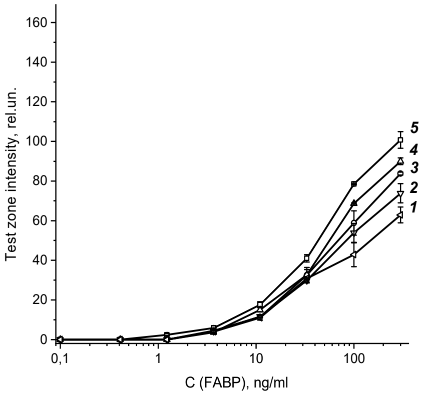

Test zone intensity, rel.un.
C (FABP), ng/ml
5
4
3
2
1

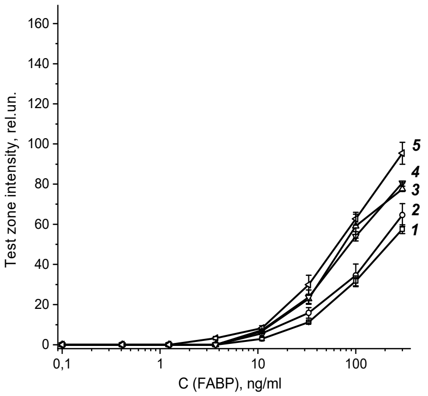

Test zone intensity, rel.un.
C (FABP), ng/ml
5
4
3
2
1

C

D

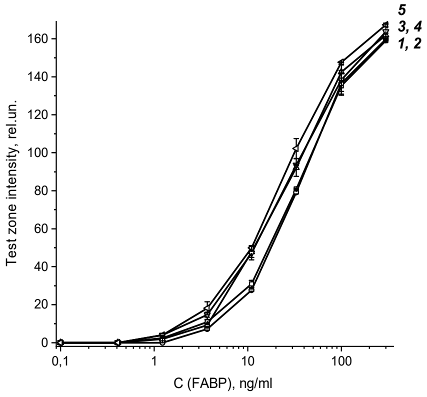

E

Figure S6 – Concentration dependences of FABP detection by LFIA test systems with conjugates GNFs 20/0.12/0.5–Ab6/FABP(0,5-20) (A), GNFs 20/0.08/0.5–Ab6/FABP(0.5-20) (B), GNFs 20/0.04/0.5–Ab6/FABP(0.5-20) (C), GNFs 20/0.12/1–Ab6/FABP(0,5-20) (D) и GNFs 20/0.12/0.2–Abs/FABP(0.5-20) (E). Curves correspond to concentrations Ab6/FABP in the synthesis of conjugates equal to 0.5 (1), 2 (2), 5 (3), 10 (4) и 20 (5) µg/mL
